# Case report: two confirmed cases of human Seoul virus infections in Indonesia

**DOI:** 10.1186/s12879-018-3482-1

**Published:** 2018-11-16

**Authors:** Khie Chen Lie, Mochamad Helmi Aziz, Herman Kosasih, Aaron Neal, Caleb Leonardo Halim, Wahyu Nawang Wulan, Muhammad Karyana, Usman Hadi

**Affiliations:** 1 Universitas Indonesia, Dr. Cipto Mangunkusumo National General Hospital, Jakarta, Indonesia; 2 INA-RESPOND, NIHRD, Ministry of Health Republic of Indonesia Building 4 (Laboratorium Terpadu), 5th Floor, Jalan Percetakan Negara No. 29, Jakarta, 10560 Indonesia; 3 National Institute of Allergy and Infectious Diseases, National Institutes of Health, Rockville, MD USA; 4 National Institute of Health Research and Development (NIHRD), Ministry of Health Republic of Indonesia, Jakarta, Indonesia; 5 Universitas Airlangga, Dr. Soetomo Hospital, Surabaya, Indonesia

**Keywords:** Seoul virus, Detection, Indonesia

## Abstract

**Background:**

Seoul virus (SEOV) is a member of hantavirus family, which is transmitted to humans by *Rattus rattus* and *Rattus norvegicus.* Diagnosing SEOV infection is difficult because the clinical presentations are often undifferentiated with other viral or bacterial infections and assays to test antibodies seroconversion and RNA detection are not available in resource-limited setting like Indonesia.

**Case presentation:**

We report two confirmed cases of SEOV infection from Indonesia. Here, we illustrate the clinical presentations, hematology and biochemistry profiles, and outcomes of the two cases. Phylogenetic analysis revealed that SEOV sequences have highest homology to isolates obtained from rodents in Indonesia.

**Conclusions:**

This report highlights the importance of considering SEOV infection in febrile patients with lymphopenia, thrombocytopenia, and elevation of liver enzyme despite the absence of hemorrhagic manifestations and renal syndromes. The public health importance of rodent-borne diseases such as SEOV infection urges an integrated epidemiological surveillance both in humans and rodents in Indonesia.

**Electronic supplementary material:**

The online version of this article (10.1186/s12879-018-3482-1) contains supplementary material, which is available to authorized users.

## Background

Seoul virus (SEOV) is a member of hantavirus family that is carried by the rodent vectors *Rattus rattus* and *Rattus norvegicus* [1]. Both rodents are widespread globally, thus making SEOV distribution worldwide [1] with confirmed human cases in Asia [2–5], Europe [6–8], and North America [9, 10]. The symptoms and signs of SEOV infection are not specific, including fever, headache, nausea, joint pain, cough, and a mild to moderate form of hemorrhagic fever with renal syndrome (HFRS) accompanied with elevation of liver enzyme [5, 10].

As these clinical presentations are undifferentiated with other infectious diseases, diagnosis can only be confirmed when IgM positive and/or rising IgG antibodies between acute and convalescent blood specimens and/or SEOV RNA in the acute specimen is detected [11]. Since the coated antigen in the commercial available antibody assay is a recombinant protein of all hantavirus species, the detected IgM/IgG antibodies are not specific to SEOV. In addition, antibody persistence could leads to false positivity, and this assay must be performed in batch to be cost-effective [11]. Molecular techniques, by SEOV RNA detection, may overcome the limitation of antibody detection, however, SEOV RNA is not detected after the acute phase. Due to the aforementioned reasons, SEOV infection in humans in Indonesia was rarely reported. Hantavirus infections based on the presence of IgM antibodies were found in Semarang [12] and Yogyakarta [13]. On the contrary, SEOV infection in rodents has been previously detected by molecular and serological methods in many areas [14, 15]. In this report, we describe two human SEOV cases diagnosed by both serologic and molecular detection in two hospitalized acute febrile illness with elevated liver enzyme without hemorrhagic manifestations and renal syndrome in Indonesia.

## Case presentation

### Methods and patients

Both patients are consented and enrolled as subjects during an observational study of Acute Febrile Illness Requiring Hospitalization (AFIRE) (NCT02763462). Clinical information and blood specimens were collected at enrollment, once during the 14–28 day period after enrollment, and at three months after enrollment. Patients who were discharged after the enrollment visit and failed to return to the hospitals for follow-up visits were seen at their homes, during which time the home environment could be casually observed. Patients’ diagnostic work-up at the hospitals (blood, respiratory, feces, or urine culture, and standard rapid diagnostic tests) and in the reference laboratory retrospectively (a panel of serological and molecular methods for the detection of bacteria and viruses) included selective testing for any culturable bacteria (i.e *Salmonella typhi*, *Streptococcus pneumonia*), *Rickettsia spp.*, *Orientia tsutsugamushi*, *Leptospira spp*., dengue, chikungunya, Zika virus, and Human Immunodeficiency Virus (HIV)*.* This study covered the cost of blood culture and other important diagnostic investigations based on the discretion of the attending clinicians at the hospitals, which may be used for clinical management.

Cases that were negative for previously described pathogens but demonstrated signs and symptoms of liver involvement or renal insufficiency were screened for hantavirus infection. Specimens from these subjects were tested for the expression of hantavirus IgM and IgG in acute and convalescent specimens (when available) by enzyme-linked immunosorbent assay (ELISA) using the Hantavirus IgM DxSelect™ and Hantavirus IgG DxSelect™ ELISA (Focus Diagnostics, Cypress, USA). Recent infection of hantavirus was confirmed if the IgM index value is > 1.10 in the acute samples and IgG index value is > 1.10 in the acute and/or convalescent samples. When serology result indicates recent hantavirus infection, acute buffy coat and plasma specimens were tested by nested reverse transcription polymerase chain reaction (nested RT-PCR) targeting the L segment of hantavirus [16] to confirm the etiology of acute febrile illness.

Viral RNA was firstly extracted from 140 μL of buffy coat and plasma-EDTA using the QIAamp® Viral RNA Mini kit (Qiagen GmbH, Hilden, Germany), according to the manufacturer’s instruction, and eluted in 60 μL of AVE elution buffer (Qiagen). RT-PCR was done in the Applied Biosystems Proflex PCR System (Thermo Fisher Scientific). The total reaction mixture volume is 25 μL, consisting of 20 μL mastermix (OneStep RT-PCR Kit (Qiagen), 1 μM each of HanL-F1 and HanL-R1 primer pair (Integrated DNA Technologies), nuclease-free water (Ambion)), and 5 μL RNA. The cycle condition of RT-PCR consists of reverse transcription (50^o^ C 30 min), initial denaturation (94^o^ C, 2 min), 35 cycles of denaturation (94^o^ C, 15 s), primer annealing (52^o^ C, 30 s), primer extension (72^o^ C, 30 s), and followed by a final extension at 72^o^ C for 10 min. The RT-PCR product was subsequently used as template for nested PCR in a 20 μL mastermix, consisting of 2X Go-Taq® Green Master Mix (Promega), 0.625 μM each of HanL-F2 and HanL-R2 primers (Integrated DNA Technologies), nuclease-free water (Ambion), and 2 μL of RT-PCR product. The nested PCR cycle condition consists of initial denaturation (95^o^ C, 5 min), followed by 25 cycles of denaturation (94^o^ C, 30 s), primer annealing (52^o^ C, 30 s), primer extension (72^o^ C, 25 s), and ended by a final extension at 72^o^ C for 8 min. The nested PCR product is visualized on a 2% agarose gel electrophoresis and imaged using the GelDoc™ EZ Imager (Bio-Rad), where positive result appears as a 412-bp band.

Positive hantavirus specimens were further tested for species-specific SEOV RT-PCR targeting the N gene of the S segment, as described by Dekonenko et al [17] The cycle condition is similar with that of hantavirus RT-PCR, but the cycle threshold was increased to 40. The reaction mixture, but replacing the HanL-F1 and HanL-R1 primer pair with the SEO-HS1 and SEO-HS2 primer pair. RT-PCR product is also visualized on 2% agarose gel electrophoresis, where positive result appears as a 250-bp band. To confirm the identity of Seoul virus, SEOV RT-PCR product (250 bp) was sequenced using the similar RT-PCR primer pair (HS1 and HS2). The sequence was then compared to the NCBI nucleotide database using BLASTn (https://blast.ncbi.nlm.nih.gov/Blast.cgi), where they aligned to nucleotides 338 through 587 of the SEOV N gene ORF. The genetic relationship was analyzed by neighbor-joining analysis using MEGA 7.0.2.

Both patients consented to have their clinical and laboratory data published and ethical clearance was approved by the IRBs of Dr. Soetomo General Hospital (192/Panke.KKE/VIII/2012) and the National Institute of Health and Research and Development (NIHRD), Ministry of Health, Indonesia (KE.01.05/EC/407/2012).

### Cases

The detailed demography, clinical, and laboratory findings are presented in Table 1. The first patient was a 55-year-old housewife, who had no history of chronic disease, visited the emergency unit of Dr. Soetomo General Hospital in Surabaya on November 2, 2015, with a 4-day history of abrupt continuous fever. On admission, she presented with fever (38 °C), lethargy, abdominal-epigastric pain, nausea, and vomiting. On examination she was alert, blood pressure was 100/60 mmHg, and pulse rate was 82 beats per minute, no unremarkable findings on systemic examination. Laboratory results revealed leukopenia, lymphopenia, thrombocytopenia and increased liver enzymes. Blood cultures and Hepatitis B Surface Antigen (HBsAg) were negative. Two days after admission body temperature returned to normal but the platelet count was still below normal range (73 × 10^3^/μL). Therefore, she was clinically diagnosed with dengue infection, treated with intravenous fluid Ringer’s lactate, and discharged 5-days after admission.

**Table 1:** Demography, clinical, and laboratory features both in hospital and reference laboratory

Patient	1	2
Age (years)	55	27
Gender	Female	Male
City	Surabaya	Jakarta
Dates of enrollment (days after fever onset)	03/11/2015 [4]	01/03/2016 [6]
Duration of hospital stay (days)	5	8
Symptoms and signs (at enrollment)		
Fever	Yes	Yes
Anorexia	No	Yes
Headache/Dizziness	No	Yes
Decrease of consciousness	No	Yes
Lethargy	Yes	No
Abdominal/Epigastric pain	Yes	No
Diarrhea	No	No
Nausea	Yes	Yes
Vomiting	Yes	Yes
Retro orbital pain	Yes	No
Arthralgia	No	Yes
Jaundice	No	Yes
Laboratory results (normal value range) (at day 1 enrollment)		
Hematocrit (40–50%)	38.8	50.6
White blood cells (3500–9000/μL)	3850	5790
Neutrophils (39.8–70.5%)	73.2	79
Lymphocyte (23.1–49.9%)	8.4	16
Platelets (150–450 × 10^3^/μL)	50,000	24,700
AST (< 41 IU/L)	216	3900
ALT (< 38 IU/L)	116	891
Blood Urea Nitrogen (BUN) (10–23 mg/dL)	10	20.3
Creatinine serum (0.5–1.2 mg/dL)	0.57	1.16
C-Reactive protein (< 5 mg/dL)	ND	41.1
Procalcitonin (< 0.5 ng/dL)	ND	5.98
Diagnostic work-up for other pathogens at reference laboratory PCR results at day 1 enrollment sample		
Dengue	Negative	Negative
Chikungunya	Negative	Negative
Zika	ND	Negative
Hantavirus	Positive	Positive
Seoul virus	Positive	Positive
Serological results (day 1 enrollment-convalescent (convalescent samples were taken 58 and 28 days after enrollment respectively for patient 1 and 2)		
Dengue IgM	Negative − Negative	Negative – Negative
Dengue IgG	Positive − Positive	Negative − Negative
Chikungunya IgG	ND − Negative	ND − Negative
Salmonella IgM	Negative − Negative	Negative − Negative
Salmonella IgG	Negative − Negative	Negative − Negative
Leptospira IgM	Negative − Negative	Negative − Negative
Leptospira IgG	Negative − Negative	Negative − Negative
Hantavirus IgM	1:800–1:400	1:200–1:3200
Hantavirus IgG	1:800–1:6400	1:400–1:12800
Murine typhus IgG	ND − Negative	ND − Negative
Scrub typhus (IgG)	ND − Negative	ND − Negative
HIV antibody	Negative − Negative	Negative − Negative

ND: Not Done

The second patient was a 27-year-old working man who visited the emergency unit of Cipto Mangunkusumo Hospital in Jakarta on March 1, 2016, with a 6-day gradual continuous fever. On admission, he presented with fever (38.7 °C), decreased level of consciousness, jaundice, anorexia, nausea, vomiting, headache, dizziness, and arthralgia. His blood pressure was 140/90 mmHg, and pulse rate was 110 beats per minute. Hematology and biochemistry results revealed lymphopenia, thrombocytopenia, and a high level of transaminases. Blood cultures, dengue virus, hepatitis A, hepatitis C, cytomegalovirus (CMV), toxoplasma, rubella, herpes simplex virus 1 (HSV-1), HSV-2, and *Salmonella* work up were negative. During 8-days of hospitalization the transaminase levels decreased over time, although they remained above normal values (AST: 102 IU/L; ALT: 134 IU/L) until discharge. Despite a negative Salmonella Typhi IgM rapid test result, the patient was diagnosed as having typhoid fever based on clinical presentation and elevated inflammatory biomarkers C-reactive protein (CRP) and procalcitonin (Table 1). He received intravenous ceftriaxone 2 × 1 g according to Ministry of Health Republic of Indonesia clinical guidelines for suspected typhoid fever cases.

**Fig. 1:**
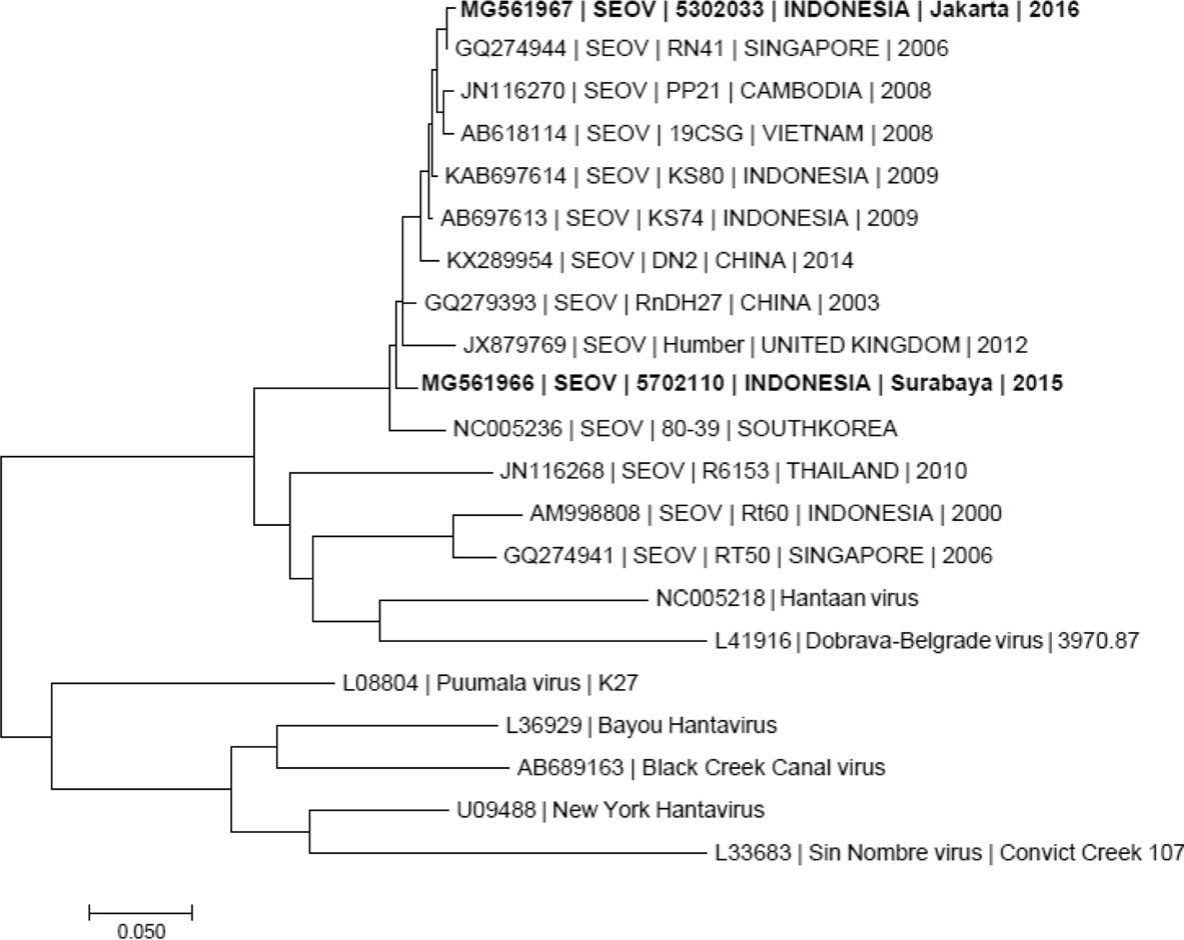
Phylogenetic analysis of the partial N gene of Seoul virus (SEOV) detected in the AFIRE study. A neighbor-joining tree was constructed based on nucleotides 338 through 587 of the SEOV N gene ORF and its homologues in other hantaviruses. The SEOV strains identified in the AFIRE study are shown in bold. The scale bar indicates nucleotide substitutions per site. The sequences of the N gene segments of SEOV obtained in this study were submitted to GenBank under accession no. MG561966 and MG561967

During follow-up visits, both patients had no remarkable symptoms and blood samples were taken. Rodent contact history was unknown for both patients upon enrollment, but during home visits we perceived that they lived in densely populated areas and close to open, rubbish-filled gutters where rodent infestations were present. However, these observations did not alter the diagnostic algorithm and were only associated with the cases after testing was completed. All serology tests were negative except for acute hantavirus infection (Table 1). The hantavirus IgM titer in patient 1 decreased in the convalescent specimen but the IgG titer increased. In patient 2, both IgM and IgG titers increased in the convalescent specimens. Since the convalescent samples were collected on different days after enrollment (days 58 and 28 for patients 1 and 2, respectively) (Additional file 1), the IgM kinetics were understandably different, with waning in patient 1 as expected. The acute samples were then tested positive for hantaviruses RNA and SEOV RNA. BLASTn analysis were performed on both samples, where sample from patient 2 was shown to be most similar (99.6% identity) to Seoul virus isolate Singapore/06(RN41) [18] (GenBank accession number GQ274944) and sample from patient 1 was shown to be most similar (98.4% identity) to Seoul virus strain Rn-DH27 (GenBank accession number GQ279393) at nucleotide 338–587 of the N gene ORF (Fig. 1).

## Discussion and conclusions

Two acute febrile illness patients from two urban cities in Indonesia, Surabaya and Jakarta, were confirmed to have infections with SEOV. This finding may suggest that previous hantavirus cases from Indonesia and other Southeast Asia countries with similar clinical presentations (elevated liver enzymes, lymphopenia, thrombocytopenia with/out hemorrhagic manifestations and renal syndromes) were SEOV infections. Consistent with previous reports, both our patients had elevated liver enzyme and thrombocytopenia [2, 6, 9, 10, 19] . Clinical misdiagnoses occurred with the two reported patients due to the non-specific signs and symptoms of SEOV infection. In the first patient, clinicians provided a diagnosis of dengue fever as she had leukopenia, thrombocytopenia, and elevated liver enzymes. In the second patient, no pathogens were detected even though extensive testing was performed. Typhoid fever was provided as the diagnosis based on clinical observations of nausea, vomiting, icterus, and decrease of consciousness, all of which may be found in severe *Salmonella typhi* infections [20]. This report highlights the importance of considering SEOV infection in febrile patients with thrombocytopenia and elevation of liver enzymes despite the absence of hemorrhagic fever with renal syndrome. Because of clinically vague presentation of SEOV infection diagnostic capabilities at hospitals in Indonesia is needed.

Unlike previous hantavirus infection reports from Southeast Asian countries [3, 4] that could not determine the infected species the cases presented here were identified as SEOV infection by molecular method. The infrequent detection of SEOV by molecular method is likely due to the short duration of viremia and the limited number of cases tested [11]. Most cases of SEOV are associated with known rodent contacts [2, 6]. Although the history of rodent contact was unknown in both patients, our phylogenetic analysis result showed high similarity (98–99%) to SEOV in Kepulauan Seribu, Jakarta, Indonesia [14], and to SEOV circulating in the region [21] isolated from rodents. This finding supports previous reports on hantavirus infection in humans in Indonesia, and further identify that SEOV was the infecting species, in line with epizoonotic evidence that SEOV [12, 15] circulates in the local rodent population [14, 15].

In summary our cases illustrate the uncommon clinical presentation of SEOV infection which is the absence of hemorrhagic manifestation and renal syndrome. Increasing hospital diagnostic capacity on SEOV will be the main challenge in resource-limited setting such as Indonesia. Although the animal reservoirs are easily found anywhere in Indonesia, the true burden of SEOV illness is not known. The public health importance of rodent-borne pathogens such as SEOV urges for integrated epidemiological surveillance both in humans and rodents in Indonesia.

## Additional file

Additional file 1: Case report timeline. (DOCX 28 kb)

## Abbreviations

AFIRE: Acute Febrile Illness Requiring Hospitalization
ALT: Alanine transaminase
AST: Aspartate transaminase
CMV: Cytomegalovirus
EDTA: Ethylenediaminetetraacetic acid
ELISA: Enzyme-linked immunosorbent assay
HFRS: Hemorrhagic fever with renal syndrome
HIV: Human Immunodeficiency virus
HSV: Herpes Simplex Virus
NIHRD: National Institute of Health and Research and Development
RNA: Ribonucleic acid
RT-PCR: Reverse transcriptase-polymerase chain reaction
SEOV: Seoul virus
SOC: Standard of care
