# Supplementary material for: Case report: two confirmed cases of human Seoul virus infections in Indonesia
Source: BMC Infect Dis. 2018 Nov 16;18:578. doi: 10.1186/s12879-018-3482-1 (PMC6240170; doi:10.1186/s12879-018-3482-1)
Supplement: Supplementary file 1 — Case report timeline. (DOCX 28 kb) [file 12879_2018_3482_MOESM1_ESM.docx]

**Patient 1 hospitalized with 4-day history of fever with no history of chronic disease.**

**Patient 2 hospitalized with 6-day history of fever.**

**Decrease of consciousness and jaundice.**

**Laboratory test: Lymphopenia, Thrombocytopenia, and Elevated Liver Enzymes.**

**Diagnosis: Typhoid Fever.**

**Treated with Ceftriaxone IV.**

**Enrolled as subject in AFIRE Study.**

**03 Nov 2015**

**Normal Physical Examination.**

**Laboratory test: Leucopenia, Lymphopenia, Thrombocytopenia, and Elevated Liver Enzymes.**

**Diagnosis: Dengue infection.**

**Treated with Ringer’s Lactate IV.**

**Enrolled as subject in AFIRE Study.**

**End date of Fever.**

**02 Nov 2015**

**04 Nov 2015**

**Discharged with improved outcome.**

**06 Nov 2015**

**Follow-up/Home visit 1.**

**No remarkable signs and symptoms.**

**31 Dec 2015**

**01 Mar 2016**

**Follow-up/Home visit 2.**

**No remarkable signs and symptoms.**

**02 Mar 2016**

**Discharged with improved outcome.**

**08 Mar 2016**

**End date of Fever.**

**11 Mar 2016**

**29 Mar 2016**

**Follow-up/Home visit 1.**

**No remarkable signs and symptoms.**

**Follow-up/Home visit 2.**

**No remarkable signs and symptoms.**

**13 Jun 2016**

**Molecular and serological analysis.**

**Molecular and serological analysis.**

**2017**

**Both patients were confirmed by positive or four-fold increase of hantavirus IgM titers, four-fold increase of hantavirus IgG titers, and Seoul virus-positive on acute samples by RT-PCR.**

**Additional File 1.** Case report timeline of two patients confirmed with SEOV infection.
